# IER3IP1 deficiency leads to increased β-cell death and decreased β-cell proliferation

**DOI:** 10.18632/oncotarget.18179

**Published:** 2017-05-25

**Authors:** Juan Sun, Decheng Ren

**Affiliations:** ^1^ Department of Medicine, The University of Chicago, Chicago, IL 60637, USA

**Keywords:** cell death and proliferation, beta-cell, IER3IP1

## Abstract

Mutations in the gene for Immediate Early Response 3 Interacting Protein 1 (IER3IP1) cause permanent neonatal diabetes mellitus in human. The mechanisms involved have not been determined and the role of IER3IP1 in β-cell survival has not been characterized. In order to determine if there is a molecular link between *IER3IP1* deficiency and β-cell survival and proliferation, we knocked down *Ier3ip1* gene expression in mouse MIN6 insulinoma cells. IER3IP1 suppression induced apoptotic cell death which was associated with an increase in Bim and a decrease in Bcl-xL. Knockdown of Bim reduced apoptotic cell death in MIN6 cells induced by IER3IP1 suppression. Overexpression of the anti-apoptotic molecule Bcl-xL prevents cell death induced by IER3IP1 suppression. Moreover, IER3IP1 also regulates activation of the unfolded protein response (UPR). IER3IP1 suppression impairs the Inositol Requiring 1 (IRE1) and PKR-like ER kinase (PERK) arms of UPR. The cell proliferation of MIN6 cells was also decreased in IER3IP1 deficient cells. These results suggest that IER3IP1 suppression induces an increase in cell death and a decrease in cell proliferation in MIN6 cells, which may be the mechanism that mutations in IER3IP1 lead to diabetes.

## INTRODUCTION

Immediate early response 3 interacting protein 1 (IER3IP1) is a highly conserved protein in different species and expressed in pancreas, heart, skeletal muscle, fetal brain cortex, kidney, liver, brain, placenta, lung and peripheral blood leukocytes [1, 2]. Human *IER3IP1* gene is located on human chromosome 18q12 and has 3 exons encoding an 82-amino acid protein. IER3IP1 contains 2 transmembrane domains and a putative G-patch domain found in several RNA-associated proteins and in type D retroviral polyproteins. IER3IP1 is localized to the endoplasmic reticulum (ER) through its C-terminal transmembrane domain [1]. IER3IP1 may be involved in transporting the proteins between the ER and the Golgi apparatus. Recently, two homozygous missense mutations in IER3IP1 were found in two unrelated consanguineous families [1]. These mutations cause a unique clinical syndrome in which affected individuals display severe infantile epileptic encephalopathy, microcephaly, simplified gyral patterns, and permanent neonatal diabetes mellitus. Molecular analysis revealed a homozygous missense mutation in exon1 of the Ier3ip1 gene, c.62T>G, causing a valine to glycine amino acid substitution at position 21(p.Val21Gly), and a second homozygous missense mutation in exon 3, c.233T>C, causing a leucine to proline substitution at position 78 (p.Leu78Pro). These observations were subsequently confirmed by another group [2]. In addition, a novel frame shift mutation p.Phe27fsSer25 and a p.Val21Gly (V21G) mutation in IER3IP1 were found in a neonatal diabetes patient [3]. The association of neonatal diabetes with IER3IP1 mutants suggests that IER3IP1 may regulate β-cell survival and/ or function.

An increase in β-cell death and a progressive β-cell mass reduction as an essential element occur during developing diabetes [4]. ER stress can induce β-cell death. A number of causes including high glucose, free fatty acids, inflammatory cytokines, hypoxia and mutations in the insulin 2 gene may induce ER stress in β-cells. ER stress is sensed by three ER transmembrane proteins: Inositol requiring 1 (IRE1), PKR-like ER kinase (PERK), and Activating transcription factor 6 (ATF6). Upon activation, these stress sensors transduce a different signaling cascade termed the unfolded protein response (UPR) to recover ER homeostasis [5]. In response to ER stress, IRE1 activates its endoribonuclease activity and splicing of transcription factor X-box protein binding 1 (sXBP1) mRNA by its oligomerization and autophosphorylation. sXBP-1 upregulates chaperone and ER-associated protein degradation (ERAD) protein expression. PERK can phosphorylate the α subunit of eukaryotic initiation factor 2 (eIF2α) leading to general protein synthesis inhibition, while increasing translation of activating transcription factor 4 (ATF4), which regulates genes important for ER homeostasis. The translocation of the processed form of ATF6 to the nucleus can upregulate UPR homeostatic effectors involved in protein folding, processing, and degradation [6]. The three arms of the UPR regulate several downstream effectors to reduce ER stress. When ER stress cannot be reduced, the UPR activates death effectors and induces β-cell apoptosis.

In this study, we will define the role of IER3IP1 in β-cell survival and proliferation, and identify the mechanism of β-cell apoptosis induced by IER3IP1 suppression.

## RESULTS

### IER3IP1 suppression leads to apoptosis in MIN6 cells

**Figure 1:**
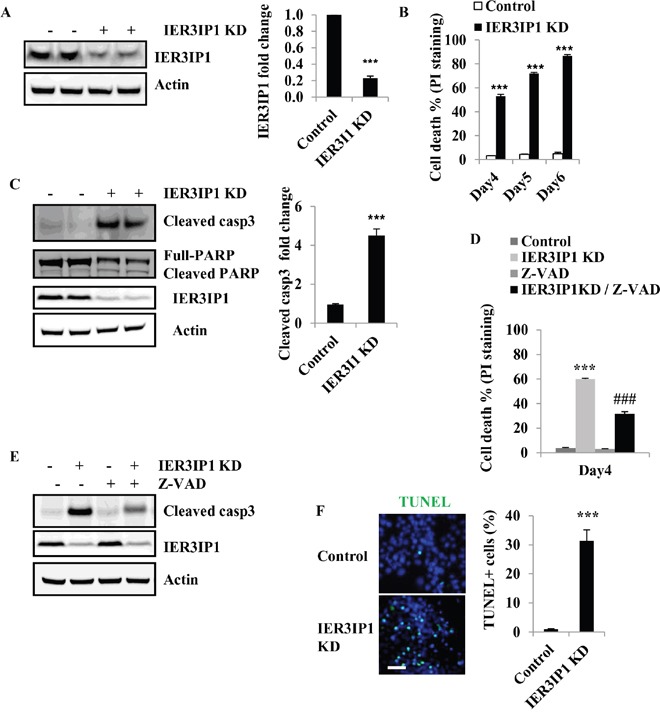
Reduced IER3IP1 expression leads to apoptosis in MIN6 cells **(A)** IER3IP1 protein levels in IER3IP1 KD MIN6 cells. MIN6 cells were infected with a lentivirus vector that drives expression of shRNA that targets the IER3IP1 transcript (IER3IP1 KD) or control lentivirus vector that does not have a specific target. IER3IP1 protein levels were measured 4 days after infection by western blot (n=3). The loaded proteins in the two “- or +” lines were the same but from the different plates. **(B)** Cell death was determined by PI-staining in MIN6 cells after infection with IER3IP1 shRNA lentivirus. **(C)** Cleaved caspase3 protein levels in IER3IP1 KD cells. 4 days after infection with IER3IP1 shRNA lentivirus, cleaved caspase3 and PARP proteins were assayed by western blot (n=3). The loaded proteins in the two “- or +” lines were the same but from the different plates. **(D)** Z-VAD inhibits caspase3 cleavage induced by IER3IP1 KD. MIN6 cells were treated with the caspase inhibitor Z-VAD (20 μM), for 2 hours prior to infection with IER3IP1 shRNA lentivirus. 4 days later, cleaved caspase3 protein was assayed by western blot (n=3). **(E)** Z-VAD inhibits cell death induced by IER3IP1 KD in MIN6 cells. MIN6 cells were treated with 20 μM Z-VAD, for 2 hours prior to infection with IER3IP1 shRNA lentivirus and cell death was determined by PI-staining on day 4 (n=3). ***P<0.001 compared to control group. ###P<0.001 compared to IER3IP1 KD group. **(F)** TUNEL labeling of IER3IP1 KD MIN6 cells. 4 days after infection with IER3IP1 shRNA lentivirus, apoptotic cells were assayed by TUNEL staining. Quantitative TUNEL data are shown. Scale bar, 20 μm. ***P<0.001 compared to control group. Values are mean ± SEM.

To investigate whether IER3IP1 regulates β-cell survival, IER3IP1 shRNA lentivirus was used to knock down (KD) expression of IER3IP1 in MIN6 cells. The mRNA levels of IER3IP1 were significantly reduced to 24 ± 1% of control cells on day 4 after infection with *lentiviral* IER3IP1 shRNA (not shown) and IER3IP1 protein levels were decreased to 22 ± 2% of control (Figure 1A). The effect of IER3IP1 KD on cell death was determined using propidium iodide (PI) staining followed by Fluorescence-activated cell sorting (FACS). After infection with IER3IP1 shRNA lentivirus, the proportion of PI staining positive cells increased from 52 ± 3% on day 4 to 72 ± 2% on day 5 and 86 ± 2% on day 6 (all *P*<0.001), respectively (Figure 1B). In addition, the cleaved caspase3 protein levels were increased more than 3-fold in IER3IP1 KD MIN6 cells compared to control cells (Figure 1C). IER3IP1 KD also induced an increase in cleaved poly (ADP-ribose) polymerase (PARP), another apoptosis marker (Figure 1C). The pan-caspase inhibitor Z-VAD significantly inhibited the increase in PI staining positive cells (Figure 1D). The PI staining positive cells were decreased from 60 ± 2% in IER3IP1 KD cells to 27 ± 1% in Z-VAD/IER3IP1 KD cells (*P*<0.001) (Figure 1D). Immunoblot analysis showed that Z-VAD significantly inhibited IER3IP1 KD-induced caspase3 cleavage (Figure 1E). To further determine the effects of IER3IP1 suppression on cell death, we use terminal dUTP nick end labeling (TUNEL) assay to measure cell apoptosis. The results showed that IER3IP1 KD significantly increased the number of TUNEL positive cells. The TUNEL-labeled cells were inceased from 1.0 ± 0.2% in control cells to 31.1 ±2.8 % in IER3IP1 KD cells on day 4 (*P*<0.001) (Figure 1F). Collectively, these findings indicate that reduced IER3IP1 expression induces apoptosis in MIN6 cells.

### IER3IP1 suppression decreases mitochondrial membrane potential and increases cytochrome c release

**Figure 2:**
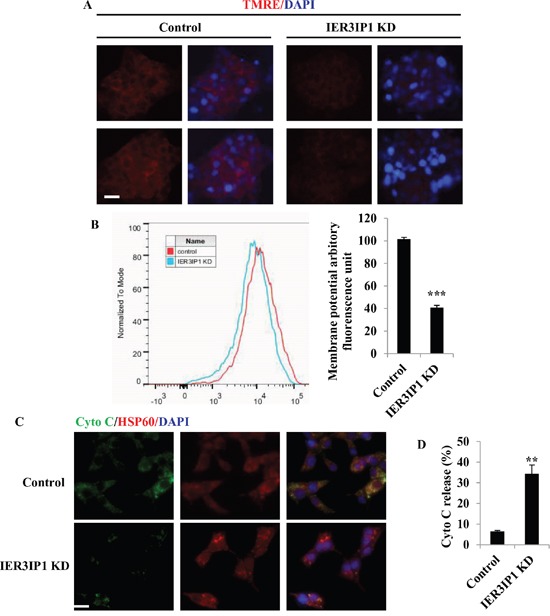
Effects of IER3IP1 suppression on ΔΨm and cytochrome c release **(A+B)** MIN6 cells were treated with IER3IP1 shRNA lentivirus for 3 days. Cells were then stained with TMRE dye to measure ΔΨ_m_. Scale bar, 20 μm. ***P<0.001. **(C+D)** IER3IP1 suppression induces cytochrome c release. 3 days after IER3IP1 KD in MIN6 cells, the images were taken under fluorescence microscopy **(C).** The green, red and blue staining represent cytochrome c, HSP60 and DAPI, respectively. Scale bar, 20 μm. The release of cytochrome c was also measured **(D).** **P<0.01 compared to control group. Values are mean ± SEM.

During apoptosis, the mitochondrial membrane potential (ΔΨ_m_) decreases [7]. To determine the effects of IER3IP1 suppression, mitochondrial membrane potential (ΔΨ_m_) was measured by quantifying the average mitochondrial fluorescence intensity of tetramethylrhodamine, ethyl ester (TMRE). TMRE uptake into mitochondria was dramatically decreased in IER3IP1 KD cells, indicating that IER3IP1 KD significantly decreased mitochondrial membrane potential (Figure 2A). The relative membrane potential fluorescence in mitochondria decreased by 60% in IER3IP1 KD cells compared to control cells (*P*<0.001) (Figure 2B). IER3IP1 KD induced an increase in cytochrome c release from mitochondria, the key event in activating apoptosis (Figure 2C). Cytochrome c release increased from 6.5 ± 0.4% in control cells to 34.4 ± 4.3% in IER3IP1 KD cells (*P*<0.001) (Figure 2D).

### IER3IP1 suppression activates Bax in MIN6 cells

**Figure 3:**
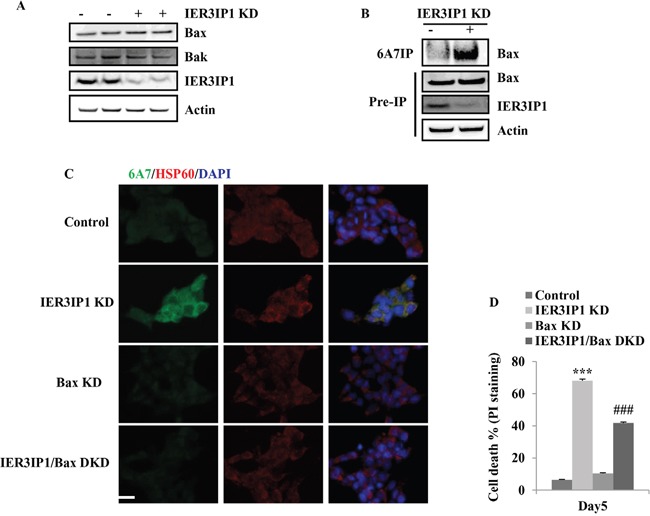
IER3IP1 suppression induces Bax activation in MIN6 cells **(A)** Bax and Bak protein levels in control and IER3IP1 KD cells. 3 days after IER3IP1 KD in MIN6 cells, Bax and Bak protein levels showed no difference between control and IER3IP1 KD cells (n=3). The loaded proteins in the two “- or +” lines were the same but from the different plates. **(B)** Immunoprecipitate of Bax. 3 days after IER3IP1 KD in MIN6 cells, cells were lysed in 1% CHAPS and then immunoprecipitated with the 6A7 anti-BAX antibody. Immunoprecipitates were analyzed by anti-BAX (N20) immunoblots. **(C)** Bax KD inhibits IER3IP1 KD-induced Bax activation. 3 days after IER3IP1 KD in MIN6 cells, the images were taken under fluorescence microscopy. Scale bar, 20 μm. **(D)** Bax KD inhibits cell death induced by IER3IP1 suppression. MIN6 cells were infected with IER3IP1 shRNA or IER3IP1/Bax double shRNA lentivirus. 4 days later, cell death was determined by PI-staining (n=3). ***P<0.001 compared to control group. ###P<0.001 compared to IER3IP1 KD group. Values are mean ± SEM.

IER3IP1 KD did not induce a significant increase in *Bax* and *Bak* mRNA levels in MIN6 cells (not shown). Western blotting also showed no increase in Bax and Bak protein levels in IER3IP1 KD cells compared to control cells (Figure 3A). Since Bax is stationed in the cytosol and can translocate into mitochondria once activated by a diversity of stress. The Bax conformational change stimulated by stress was examined using the monoclonal antibody 6A7, which only recognizes the N-terminal epitope of Bax [8]. The results showed that the amount of Bax precipitated by 6A7 anti-Bax antibody was increased in IER3IP1 KD cells (Figure 3B). Bax translocation to mitochondria induced by IER3IP1 KD was also increased (Figure 3C). Bax KD inhibited the increase of Bax translocation to mitochondria (Figure 3C). The PI staining positive cells (cell death) significantly decreased from 68.0 ± 1.7% in IER3IP1 KD cells to 41.8 ± 4.3% in IER3IP1/Bax DKD cells (*P*<0.001) (Figure 3D).

### Bim is increased following IER3IP1 suppression and contributes to cell apoptosis induced by IER3IP1 suppression

**Figure 4:**
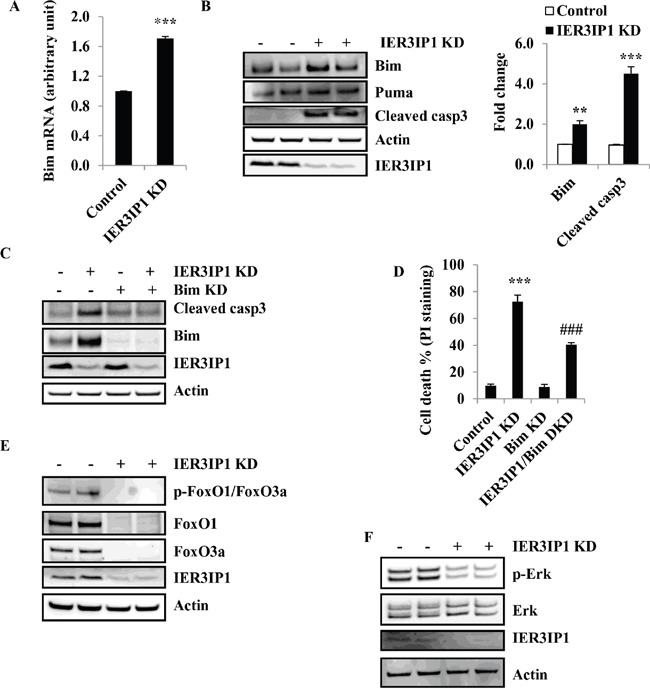
Bim is increased in MIN6 cells following IER3IP1 suppression **(A)** Bim mRNA levels in control and IER3IP1 KD cells. 4 days after IER3IP1 KD in MIN6 cells, Bim mRNA levels were measured by real time quantitative reverse transcription-PCR (qRT-PCR) in MIN6 cells (n=3). ***P<0.001 compared to control group. Values are mean ± SEM. **(B)** Bim and Puma protein levels in IER3IP1 KD cells. 4 days after IER3IP1 KD in MIN6 cells, immunoblot analysis was performed to determine Bim, Puma and cleaved caspase3 protein levels in IER3IP1 KD MIN6 cells. The bar graph depicts the relative changes in the levels of the indicated proteins using densitometry analysis of the Western blots (n=3). Values are mean ± SEM. The loaded proteins in the two “- or +” lines were the same but from the different plates. **(C)** Western blot of IER3IP1/Bim DKD cells. 4 days after IER3IP1/Bim DKD in MIN6 cells, immunoblot of Bim and cleaved caspase3 in cells. **(D)** Measurement of cell death. 4 days after Bim/IER3IP1 DKD in MIN6 cells, cell death was determined by PI-staining (n=3). ***P<0.001 compared to control group. ###P<0.001 compared to IER3IP1 KD group. Values are mean ± SEM. **(E+F)** Western blot of IER3IP1 KD MIN6 cells. 4 days after IER3IP1 KD in MIN6 cells, phosphorylation of FoxO1/FoxO3a, FoxO1, FoxO3a **(E)**, p-Erk and Erk **(F)** were determined by Western blot. The loaded proteins in the two “- or +” lines were the same but from the different plates.

Since Bcl-2 family members are widely involved in apoptotic cell death, the mRNA levels of pro-apoptotic Bcl-2 family members were determined by real-time quantitative PCR. Indeed, IER3IP1 KD resulted in an increase in Bim mRNA levels by 70% (P<0.001) (Figure 4A). Western blotting showed a corresponding increase in Bim protein levels by 100% (*P*<0.01) and a 2.5 - fold increase in cleaved caspase3 protein (*P*<0.001), respectively. However, Puma protein levels were unchanged in IER3IP1 KD cells (Figure 4B).

To determine whether Bim up-regulation is the key factor for cell death induced by IER3IP1 suppression, we knock down Bim by the lentiviral shRNA in MIN6 cells. The results showed that Bim protein levels were significantly decreased in Bim KD cells compared to control cells (Figure 4C). The increase in cleaved caspase3 was also inhibited in Bim/IER3IP1 double knockdown (DKD) cells compared to IER3IP1 KD cells alone (Figure 4C). Following IER3IP1 knockdown, 72.4 ± 4.9% of the MIN6 cells took up the PI stain, while in the Bim/IER3IP1 DKD group, only 40.5 ± 2.5% (*P*<0.001 compared to IER3IP1 alone) took up the PI stain indicative of a 32% decrease in dead cells (Figure 4D). Since Bim is able to be regulated at the transcriptional level by FoxO1 and FoxO3a and the post-translational level by Erk, we determined whether FoxO1, FoxO3a and Erk are involved in Bim upregulation induced by IER3IP1 KD. *Unexpectedly*, both FoxO1 and FoxO3a were decreased in IER3IP1 KD cells compared to control cells (Figure 4E). Moreover, phosphorylation of Erk were also decreased in IER3IP1 KD cells (Figure 4F). The results indicate that Bim upregulation in IER3IP1 KD cells is not mediated by an increase in the expression of FoxO1, FoxO3a and Erk.

### Bcl-xL prevents Bim increase and reduces cell death in IER3IP1 KD MIN6 cells

**Figure 5:**
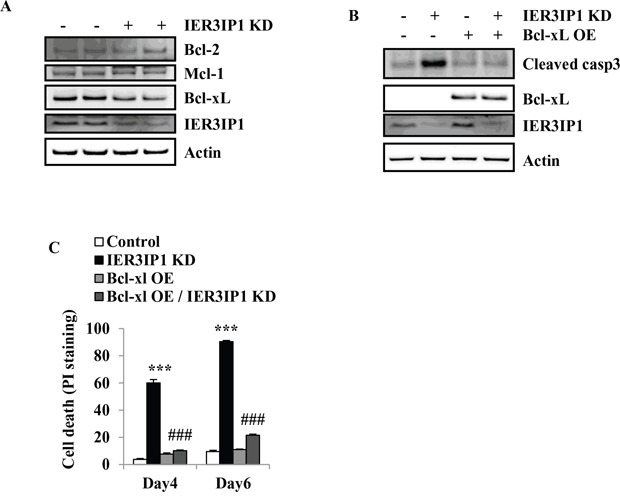
Overexpression of Bcl-xL reduces cell death induced by IER3IP1 suppression **(A)** Protein levels of anti-apoptotic Bcl-2 family members in IER3IP1 KD cells. 4 days after infection with IER3IP1 shRNA lentivirus, protein levels of Bcl-2, Bcl-xL and Mcl-1 in MIN6 cells were analyzed by Western blot. The loaded proteins in the two “- or +” lines were the same but from the different plates. **(B)** Effect of overexpression of Bcl-xL on the cleavage of caspase3. MIN6 cells were infected with IER3IP1 shRNA lentivirus and retrovirus overexpressing Bcl-xL for 3 days, then protein levels of cleaved caspase3 and Bcl-xL were determined by Western blot. **(C)** Cell death was determined by PI-staining. MIN6 cells were infected with IER3IP1 shRNA lentivirus and Bcl-xL-overexpressed retrovirus, cell death was determined by PI-staining on day 4 and day 6 (n=3). ***P<0.001 compared to control group. ###P<0.001 compared to IER3IP1 KD group. Values are mean ± SEM.

To determine whether IER3IP1 KD can also induce changes in anti-apoptotic Bcl-2 family members, the protein levels of Bcl-2, Bcl-xL and Mcl-1 were determined. The results showed that Bcl-xL protein levels were decreased in IER3IP1 KD cells. IER3IP1 KD did not change the protein levels of Bcl-2 and Mcl-1 (Figure 5A). To determine whether increasing Bcl-xL expression can inhibit IER3IP1 KD-induced cell death, Bcl-xL was overexpressed in MIN6 cells. The results showed that Bcl-xL inhibited the increase in cleaved caspase3 protein induced by IER3IP1 suppression (Figure 5B). Overexpression (OE) of Bcl-xL also decreased the enhanced cell death in IER3IP1 KD cells. Following overexpression of Bcl-xL, the cells staining positive for PI decreased from 90.4 ± 0.9% in IER3IP1 KD cells to 21.6 ± 0.8% (*P*<0.001) in IER3IP1 KD/Bcl-xL OE cells on day 6 (Figure 5C). Taken together, these findings demonstrate that the increase of Bim induces cell death in IER3IP1 KD MIN6 cells and this effect is inhibited by Bcl-xL OE.

### IER3IP1 suppression decreases UPR activation

**Figure 6:**
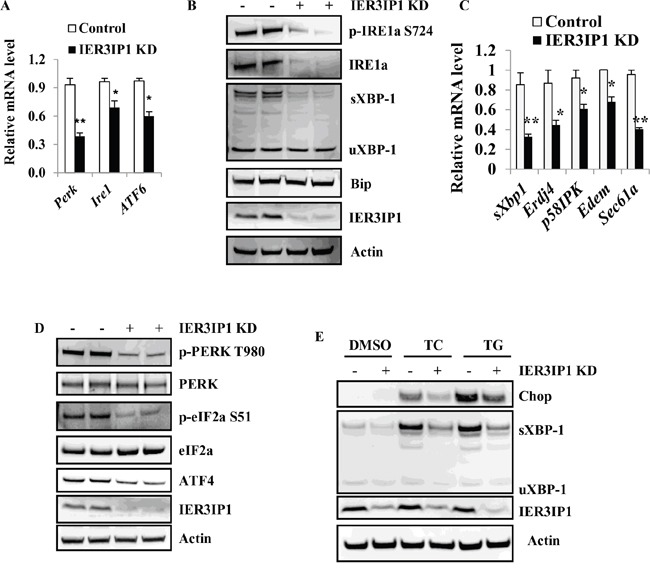
IER3IP1 suppression decreases the UPR activation **(A)** mRNA levels of IRE1α, Perk and Atf6 in IER3IP1 KD cells. 4 days after infection with IER3IP1 shRNA lentivirus, mRNA levels of IRE1α, Perk and Atf6 in IER3IP1 KD cells were determined by qRT-PCR. *P<0.05, **P<0.01 compared to control group. Values are mean ± SEM. **(B)** Effect of IER3IP1 suppression on the IRE1α arm of the UPR. 4 days after IER3IP1 KD in MIN6 cells, phosphorylation of IRE1α, Bip, sXBP-1 and uXBP-were determined by Western blot. The loaded proteins in the two “- or +” lines were the same but from the different plates. **(C)** mRNA levels of downstream targets of sXBP-1 in IER3IP1 KD cells. 4 days after IER3IP1 KD in MIN6 cells, mRNA levels of downstream targets of sXBP-1, Erdj4, p58IPK, EDEM and Sec61a, were determined by qRT-PCR. Values are mean ± SEM. **(D)** Effect of IER3IP1 suppression on the Perk arm of the UPR. 4 days after IER3IP1 KD in MIN6 cells, phosphorylation of Perk and eIF2a, and ATF4 were determined by Western blot. The loaded proteins in the two “- or +” lines were the same but from the different plates. **(E)** IER3IP1 KD decreases UPR activation induced by ER stress in MIN6. 4 days after IER3IP1 KD, MIN6 cells were treated with 10 μg/ml tunicamycin (TC) or 1 μM thapsigargin (TG) for 6 hours. Then the expression levels of Chop, sXBP-1, uXBP-1 and IER3IP1 were determined by Western blot.

Since IER3IP1 protein is present in the ER, we determined whether IER3IP1 suppression can cause ER stress and induce the UPR. Surprisingly, mRNA levels of IRE1α, Perk and ATF6, were significantly decreased in IER3IP1 KD cells compared to control cells (P<0.01 or P<0.05) (Figure 6A) indicating that UPR activation is decreased in IER3IP1 KD cells. To further confirm the role of IER3IP1 in UPR activation, the levels of UPR signaling proteins were measured. Both phosphorylation and protein levels of IRE1α were dramatically decreased (Figure 6B). The expression of spliced XBP-1, a downstream target of IRE1α, was reduced with IER3IP1 KD (Figure 6B). Moreover, mRNA levels of downstream effectors of sXBP-1 including Erdj4, p58IPK, EDEM and Sec61a, were all significantly decreased (P<0.01 or P<0.05) (Figure 6C). In addition, although protein levels of PERK were unchanged, phosphorylation of PERK was dramatically decreased (Figure 6D). Phosphorylation levels of eIF2a and protein levels of ATF4, two downstream targets of PERK, were decreased (Figure 6D). Unfortunately, ATF6 proteins were undetectable using the commercial antibodies in MIN6 cells. To further determine the effect of IER3IP1 suppression on UPR activation, the expression of spliced XBP-1 protein induced by ER stressors TC and TG was measured in IER3IP1 KD MIN6 cells. Tunicamycin (TC, induces ER stress by inhibiting N-linked glycosylation) and thapsigargin (TG, induces ER stress by inhibiting sarco/endoplasmic reticulum calcium ATPase) significantly increased spliced XBP-1 and Chop in control cells (Figure 6E). However, compared to control groups, IER3IP1 KD significantly inhibited the increase in protein levels of sXBP-1 and Chop induced by TC and TG treatment (Figure 6E). These data suggest that IER3IP1 decreases UPR activation at least through shutting down the IRE1α and PERK arms of the UPR.

### IER3IP1 suppression decreases cell proliferation in MIN6 cells

**Figure 7:**
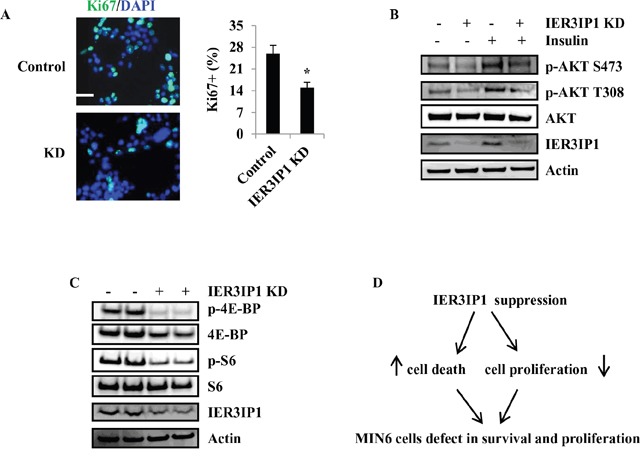
IER3IP1 suppression decreases MIN6 cells proliferation **(A)** Ki-67 staining of MIN6 cells. 2 days after IER3IP1 KD in MIN6 cells, Ki-67 staining for proliferation cell nuclei was performed in MIN6 cells. The Ki-67 staining was shown in the left. Scale bar, 20 μm. Quantitative Ki-67 staining data were shown in the right. Values are means ± SEM, *P < 0.05 compared to control cells. **(B+C)** Protein levels of phosphorylation of AKT, Erk, 4E-BP and S6. 4 days after IER3IP1 KD in MIN6 cells, phosphorylation of AKT **(B)**, 4E-BP and S6 **(C)** were determined by Western blot. The loaded proteins in the two “- or +” lines were the same but from the different plates. **(D)** Model presents how IER3IP1 suppression affects MIN6 cells. IER3IP1 suppression induces an increase in cell death and a decrease cell proliferation in MIN6 cells.

Besides cell survival, we also determine whether IER3IP1 regulates cell proliferation. The proliferative marker Ki67 staining showed that proliferation of cells was decreased by 42% in IER3IP1 KD cells compared with control cells (Figure 7A). To elucidate the mechanism by which IER3IP1 modulates cell proliferation, we analyzed the proteins related to cell proliferation including protein kinase B (PKB, AKT), 40S ribosomal protein S6 (S6) and eukaryotic initiation factor 4E (eIF4E) binding protein (4E-BP) [9, 10]. Phosphorylation levels of Akt at Ser-473 (p-AKT S473) and Thr-308 (p-AKT T308) were decreased in IER3IP1 KD cells (Figure 7B). IER3IP1 suppression also induced a decrease in protein levels of p-4E-BP and p-S6 (Figure 7C). Taking the results together, we can conclude that both AKT and mTOR signaling pathways are involved in IER3IP1-regulated cell proliferation. Our working model suggests that IER3IP1 suppression induces an increase in cell death and a decrease in cell proliferation in MIN6 cells (Figure 7D).

## DISCUSSION

IER3IP1 is thought to be involved in the endoplasmic reticulum stress response [1]. Clinical studies have demonstrated that neonates with mutations in IER3IP1 develop early-onset permanent diabetes [1, 2]. An autopsy specimen from one patient showed increased apoptosis in pancreatic β-cells indicating that β-cell death may be the pathogenetic mechanism for the neonatal diabetes in this syndrome [1]. However, the role of IER3IP1 in β-cells and how IER3IP1 deficiency causes β-cell death are unclear. The present studies were undertaken to define the molecular mechanisms responsible for cell death induced by IER3IP1 suppression. The results demonstrate that IER3IP1 suppression in MIN6 cells induces apoptotic cell death detected by caspase3 cleavage, increased TUNEL labeling and Bim upregulation. Bim is a BH3-only molecule that is essential for mitochondrial-dependent apoptosis and for inducing cell death in different cell types including neurons, T and B lymphocytes and macrophages [11–15]. Our recent studies have demonstrated that Bim is increased in Pdx1 deficient β-cells, and Bim, Puma and Bax are required for β-cell apoptosis induced by Pdx1 deficiency [16, 17]. The present experiments demonstrated that Bim KD significantly reduced IER3IP1 suppression-induced cell death. Furthermore, we demonstrated that IER3IP1 suppression induces Bax activation. Our previous studies showed that Bim can directly activate Bax and induce Bax translocation to the mitochondria leading to cell apoptosis [8, 18]. Cells survive or undergo death is decided by the balance between pro-apoptotic and anti-apoptotic factors. In IER3IP1 KD MIN6 cells, the anti-apoptotic molecule Bcl-xL was significantly decreased while Mcl-1 and Bcl-2 were unchanged. Overexpression of the anti-apoptotic molecule Bcl-xL can prevent cell death induced by IER3IP1KD. Since Bcl-xL can bind to Bim and sequester it, the decrease of Bcl-xL causes Bim release from Bcl-xL and mediates β-cell apoptosis in IER3IP1 KD cells. The mechanism by which IER3IP1 suppression induces the reduction of Bcl-xL needs to be further investigated.

Bim is expressed in the acinar cells, endocrine cells of the islets and the columnar epithelial cells in mouse. Bim is also expressed in β-cells of human islets [19]. Misregulation of Bim in β-cells is related to type 1 and 2 diabetes [20, 21]. Bim activity can be regulated through transcriptional and posttranslational mechanisms. Forkhead transcription factors can increase Bim transcription [22, 23]. As for posttranslational regulation, Bim can be phosphorylated by Erk1/2 (extracellular signal-regulated kinase 1/2) resulting in its ubiquitylation, proteasomal targeting and degradation [24, 25]. For example, transcriptional regulation of Bim by FoxO3a mediates hepatocyte lipoapoptosis [22]. To determine whether FoxO1 or FoxO3a mediates Bim upregulation induced by IER3IP1 suppression, the protein levels of FoxO1or FoxO3a were determined in IER3IP1 KD cells. Our results showed that both FoxO1 and FoxO3a were decreased in IER3IP1 KD cells, suggesting that mRNA upregulation of Bim is not mediated by FoxO1 and FoxO3a. Our data further showed that Erk is also not involved in regulating Bim expression. Since IER3IP1 is located in the ER and ER plays a key role in maintaining calcium homeostasis, IER3IP1 suppression may interrupt calcium homeostasis. Studies showed that calcium could regulate Bim-mediated cell death and Bim-deficient retinal explants are resistant to calcium overload [26]. Thus whether calcium regulates Bim and the mechanism by which Bim is upregulated need to be further explored.

Multiple factors can disturb ER homeostasis and cause accumulation of unfolded proteins, which will eventually trigger the UPR [27]. The initial effect of the UPR is to reestablish homeostasis and recover ER function by activation of transcriptional programs to induce expression of genes that are capable of enhancing the protein folding capacity of the ER and genes for ERAD. When the UPR fails to compensate, ER stress is not mitigated and homeostasis is not restored, cell death is induced, typically by apoptosis. In this study, at least two arms of UPR, including IER1α-sXBP-1, and PERK-eIF2a and -ATF4, are decreased in IER3IP1 KD cells. Furthermore, even in MIN6 cells treated with ER stressors, IER3IP1 suppression shuts down activation of the UPR. The loss of PERK expression in humans and mice increases β-cell apoptosis due to a failure of UPR regulation [28, 29]. IRE1α-sXBP-1 signaling pathway also appears to be important for regulating insulin biosynthesis, β-cell survival and proliferation [30]. The transcriptional targets of sXBP1 are highly enriched for ERAD factors, ER chaperones, and enzymes [31]. The decrease in sXBP-1 in IER3IP1 KD cells therefore reduces the ER's capacity for ERAD. The two arms of UPR defect in IER3IP1 KD cells lead to the increase in ER stress and cell death. Furthermore, ER is an important organ to maintain Ca^2+^ homeostasis. IER3IP1 deficiency in ER may disrupt Ca^2+^ homeostasis. C*ellular* Ca^2+^ overload or Ca^2+^ leakage from the cells can cause cell death [32]. However, the mechanism by which IRE3IP1 maintains ER homeostasis is unclear and whether IER3IP1 regulates Ca^2+^ homeostasis need to be investigated. In addition, ATF4 and Chop can regulate Bim expression, however, in this study both ATF4 and Chop decreased, which suggests that Bim expression is also ATF4- and Chop- independent. Whether there is a crosstalk between UPR inactivation and Bim upregulation or a decrease of Bcl-xL needs to be further studied.

Since PI3K/AKT/mTOR was the most relevant pathway relating to β-cell proliferation, survival, and cell growth [33, 34], the effects of IER3IP1 KD on AKT/mTOR signaling pathways were determined in this study. We observed a significant decrease in protein levels of p-AKT, p-4E-BP and p-S6, which was consistent with the reduction in cell proliferation in IER3IP1 KD MIN6 cells. In addition, the reduction in p-Erk may be related to the decrease in cell proliferation rather than Bim regulation. Furthermore, PERK-deficient mice exhibit severe defects in fetal/neonatal β cell proliferation and differentiation, resulting in low β-cell mass [29]. Since IER3IP1 KD induced a decrease in PERK, the reduction of PERK may also contribute to the decrease in cell proliferation in MIN6 cells.

In conclusion, we have shown that IER3IP1 suppression leads to apoptotic cell death. Bim upregulation and the reduction of Bcl-xL play critical roles in mediating apoptosis in MIN6 cells. Moreover, FoxO1, Fox3a, Erk and Chop are not involved in Bim up-regulation in IER3IP1-suppressed cells, and IER3IP1 is required for UPR activation and cell proliferation. These results suggest Bim, Bcl-xL and UPR may be the targets for therapeutic interventions in diabetes associated with mutations in IER3IP1. IER3IP1 may be a potential target for pancreatic cancer treatment.

## MATERIALS AND METHODS

### MIN6 cell culture

Mouse MIN6 insulinoma cells were cultured in Dulbecco's modified Eagle's medium supplemented with 15% FBS, antibiotics (100 units/ml penicillin and 100 μg/ml streptomycin), 1 mm sodium pyruvate, 10 mm HEPES, and 50 μm β-mercaptoethanol. The cells were maintained at 37°C in an atmosphere of 5% CO_2_ and 100% humidity.

### Quantitation of cell death

Cell death was quantified by propidium iodide (PI) staining followed by flow cytometric analyses (FACS) using a FACS Caliber (BD Bioscience) and FlowJo software [35]. Propidium iodide intercalates into double-stranded nucleic acids. PI is excluded by viable cells but can penetrate membranes of dying or dead cells. 20 μM Z-VAD-FMK (carbobenzoxy-valyl-alanyl-aspartyl-[Omethyl]-fluoromethylketone) was added to the medium 2 hours prior to treatment of MIN6 cells by IER3IP1 shRNA lentivirus. Z-VAD was added to the cells on day1 and day3.

### Ki67 staining

Cell proliferation was assessed by Ki67 staining. 3 days after IER3IP1 KD, MIN6 cells were stained with Ki67-FITC (652409, BioLegend) according to the manufacturer's instructions. In brief, cells were washed with PBS, fixed in 70% ethanol at 20°C for 1 hour, then washed 3 times with BioLegend Cell Staining Buffer and resuspend the cells at the concentration of 0.5-10 × 10^6^/ml. Cell suspension was mixed with FITC-conjugated Ki-67 antibody and incubated at room temperature in the dark for 30 minutes. Then cells were washed 2 times with BioLegend Cell Staining and resuspend in 0.5 ml cell staining buffer for flow cytometric analysis.

### Quantification of mRNA levels

RNA isolation, first strand cDNA synthesis, and TaqMan gene expression assays were performed as previously described [16]. RNA levels were normalized to the amount of actin mRNA in the same sample. Applied Biosystems (Foster City, CA) TaqMan assay numbers were: Mouse actin B, 4352933; IER3IP1, Mm01263577_g1; Bim, Mm00437796_m1 and Puma, Mm00519268_m1.

### Retrovirus infection

Human Bcl-xL was cloned into the retroviral expression vector MSCV-IRES-GFP (pMIG) (Addgene). The production of amphotropic retroviruses using the 293GPG packing cell line was performed as described previously [16]. Retroviral plasmids were transfected using FuGene6 (Roche) according to the manufacturer's protocols. Retroviral transduction of each indicated BCL-2 family protein was confirmed by western blotting. MIN6 cells were infected with these retroviralvectors at multiplicity of infection (MOI) of 10. Three days after infection with Bcl-xL retrovirus, MIN6 cells were lysed and probed with antibody for immunoblot analysis.

### Lentivirus-mediated shRNA expression

The pLKO.1-puro lentiviral vector was generously provided by Dr. Sheila Stewart of Washington University Medical School (St. Louis, MO). Potential shRNA targets in the murine IER3IP1 mRNA (thermal Scientific) were identified by quantitative reverse transcriptase PCR (QRT-PCR). Recombinant lentiviral particles were prepared by transfecting HEK 293T cells with the appropriate pLKO.1-puro plasmid plus pHR′CVM8.2 delta R and pCMV-VSV-G plasmids. Lentivirus was added to the medium on day 1.

### Western blot

MIN6 cells were lysed in cell lysis buffer containing 1% Triton X-100, 1 mm EDTA, 1 mm EGTA, 10 mm dithiothreitol, 1 mm Na_3_VO_4_, and complete protease inhibitor mixture. Equal amounts of protein were resolved by 10% or 4–12% NuPAGE (Invitrogen) gels, transferred onto PVDF membrane (Millipore), and blots were probed with antibodies against IER3IP1 (sc-84849; Santa Cruz), Puma (7467; Cell Signaling Technologies), Bim (202000; Calbiochem), cleaved caspase-3 (9661; Cell Signaling Technologies), p-FoxO1/Fox3a (9461; Cell signaling), FoxO1 (2880; Cell signaling), Fox3a (2497; Cell signaling), β-actin (A-2066; Sigma), Bax (6A7) (2281-MC-100; Trevigen), Bax (N20) (sc-493, Santa Cruz), Bak (upstate, 06536), p-IRE1α (NB100-2323; Novus), IRE1α (3294; Cell signaling), p-PERK (3179, Cell signaling), PERK (sc-9477; Santa Cruz), ATF4 (ARP37017_P050, Aviva systems biology); p-eIF2a (9721, Cell signaling), eIF2a (9722; Cell signaling), and Chop (2895; Cell signaling). To detect BAX activation, immunoprecipitation (IP) was performed using 1% CHAPS buffer (1% CHAPS, 142.5 mM KCl, 2 mM CaCl_2_, 20 mM Tris-Cl, pH 7.4). Anti-6A7 IP was performed using 1% CHAPS buffer. Antibody detection was accomplished using enhanced chemiluminescence (PerkinElmer) and LAS-3000 Imaging system (FUJIFILM).

### Flow cytometric analysis of mitochondrial membrane potential

Mitochondrial membrane potential was assessed by TMRE (tetramethylrhodamine, ethyl ester) staining followed by flow cytometric analysis [36]. TMRE enters cells and reversibly accumulates in the highly negatively charged mitochondrial matrix according to the Nernst equation, allowing the potential to be measured.

### Immunofluorescence analysis of cytochrome c

After 3 days treatment with lentiviral control or IER3IP1 shRNA, MIN6 cells were fixed for 15 min in 4% paraformaldehyde, permeabilised with 0.5% Triton X-100/PBS for 5 min and then incubated for 1 h in a 5% BSA/PBS blocking solution. Then cells were incubated for overnight at 4°C with a mouse monoclonal anti-cytochrome c IgG (Pharmingen) followed by exposure to a goat anti-mouse Alexa488-conjugated secondary antibody (invitrogen). Images were obtained using an Evos microscope (Advanced microscope group). The release of cytochrome C was measured by ELISA kit (Abcam).

### TUNEL staining

The apoptotic cell death was determined by the terminal deoxynucleotidyltransferase-mediated dUTP nick end labeling (TUNEL) labeling (Promega Corp) according to the manufacturer's instructions.

### Statistical analysis

The 2-tailed unpaired Student's t test was used to assess the statistical significance of differences between 2 sets of data. Differences were considered significant when P < 0.05. In all experiments, the number of asterisks is used to designate the following levels of statistical significance: *** P < 0.001, **P < 0.01, *P < 0.05 compared with control group. #### P < 0.001, ##P < 0.01, #P < 0.05 compared with IER3IP1 KD group. Results are presented as mean ± SEM. The data are representative experiments from another two with very similar results.

This study was supported by P30-DK020595 (The University of Chicago and Diabetes Research and Training Center).
